# Effect of electronic reminders on patients’ compliance during clear aligner treatment: an interrupted time series study

**DOI:** 10.1038/s41598-022-20820-5

**Published:** 2022-10-05

**Authors:** Lan Huong Timm, Gasser Farrag, Daniel Wolf, Martin Baxmann, Falk Schwendicke

**Affiliations:** 1 Sunshine Smile GmbH, Windscheidstraße 18, 10627 Berlin, Germany; 2 Orthodentix, Arnoldstrasse 13 b, 47906 Kempen, Germany; 3 Department of Oral Diagnostics, Digital Health and Health Services Research, Charité – Universitätsmedizin Berlin, Aßmannshauser Straße 4-6, 14197 Berlin, Germany; 4 DrSmile - DZK Deutsche Zahnklinik GmbH, Königsallee 92a, 40212 Düsseldorf, Germany

**Keywords:** Dentistry, Patient education

## Abstract

Patient compliance is relevant to achieving therapeutic goals during clear aligner therapy (CAT). The aim of this study was to evaluate the efficacy of remote electronic (e-)reminders and e-feedback on compliance during CAT using an interrupted time series (ITS) analysis. We used routinely collected mobile application data from a German healthtech company (PlusDental, Berlin). Our primary outcome was self-reported compliance (aligner wear time min. 22 h on 75% of their aligners were classified as fully compliant, min. 22 h on 50–74.9% of their aligners: fairly compliant; min. 22 h on < 50% of their aligners: poorly compliant). E-reminders and e-feedback were introduced in the 1st quarter of 2020. Compliance was assessed at semi-monthly intervals from June-December 2019 (n = 1899) and June-December 2020 (n = 5486), resulting in a pre- and post-intervention group. ITS and segmented regression modelling were used to estimate the effect on the change in levels and trends of poor compliance. Pre-intervention, poor compliance was at 24.47% (95% CI: 22.59% to 26.46%). After the introduction of e-reminders and e-feedback (i.e., post-intervention), the percentage of poorly compliant patients decreased substantially, levelling off at 9.32% (95% CI: 8.31% to 10.45%). E-reminders and e-feedback were effective for increasing compliance in CAT patients.

*Clinical Significance*: Orthodontists and dentists may consider digital monitoring and e-reminders to improve compliance and increase treatment success.

## Introduction

Patient compliance during orthodontic therapy (e.g., towards wearing a removable appliance, attending regular re-evaluation and adaptation visits, adhering to specific oral hygiene requirements) has been shown highly relevant to achieving therapeutic goals and reducing adverse effects^1–4^. Both chairside approaches (oftentimes involving written information, videos, or teaching aids, and demanding significant chairside time, i.e. generating considerable costs)^5^ and remote interventions (e.g. telephone, text, or online reminders, which are scalable and come at high accessibility) have been suggested to improve compliance^5–8^; the latter have been proven to improve patient compliance in healthcare in general^9–11^. Considering the widespread use of smartphones globally, remote interventions through messages and mobile applications offer a viable, low-cost, and equitable strategy to improve compliance^4,11^.

Studies have shown that mobile short messaging could have a positive impact on short-term behavioural outcomes^12^ and that active reminders (weekly text messaging) for patients undergoing orthodontic treatment could improve oral health^13^. Weekly reminders were also found to improve the memorability of the information given by orthodontists in the office, increasing compliance^3^.

During clear aligner treatment (CAT), electronic messaging and self-management or regular notifications by the treating dentist using mobile applications may improve patients' understanding of their therapy, their compliance, and the resulting outcomes. The efficacy of such remote electronic reminders on compliance during CAT, however, remains unclear.

Using the interrupted time-series (ITS) approach, we aimed to measure the impact of active electronic (e-)reminders and automatic e-feedback on patient compliance during CAT. The objectives of the present study were (1) to analyse patient compliance before and after the introduction of an e-reminder system during CAT, and (2) to evaluate whether the effects of this reminder system varied by gender and age.

## Materials and methods

### Study design and sample

An ITS analysis was conducted to evaluate the effect of using e-reminders and e-feedback on patient compliance during CAT. Data were collected by PlusDental, a brand of the Sunshine Smile GmbH (Berlin, Germany), during the course of routine treatment of patients, and were anonymized for research use. The study was conducted according to the guidelines of the Declaration of Helsinki. The data were collected retrospectively as a part of the treatment and anonymized for research use, which according to the Berlin State Hospital Act (Landeskrankenhausgesetz Berlin) and the recommendations of the Datenschutz und IT-Sicherheit im Gesundheitswesen (DIG) task force of the German Association for Medical Informatics, Biometry, and Epidemiology (GMDS) does not require approval from an ethics committee.

All patients were instructed to report each aligner change as well as the daily aligner wearing time using the app-based questionnaire as described in detail by Timm et al.^14^. The aligner change interval depended on the prescribed wear protocol, either after every 7 days or after 14 days of wear. Hence, reporting of changing aligners and average wear time were provided every 1–2 weeks.

We classified patients into fully, fairly and poorly compliant. Aligner wear time of ≥ 22 h with ≥ 75% of aligners and consistent use of the mobile application for aligner check-in were classified as full compliance. Patients with inconsistent app use were classified as either fairly compliant or poorly compliant based on aligner wear time: Aligner wear time of ≥ 22 h with 50–74.9% of aligners was classified as fair compliance, and aligner wear time of ≥ 22 h with only < 50% of aligners was classified as poor compliance. Thus, patients who provided no or inadequate information about their compliance would be classified as poorly compliant.

The implementation of e-reminders and automatic e-feedback was our intervention. The implementation occurred at the beginning of 2020, i.e., as of the 1st quarter of 2020 onwards these reminders were used (see below). The reporting of the study follows the STrengthening the Reporting of OBservational studies in Epidemiology (STROBE) checklist; the employed methodology is in accordance with the World Medical Association Declaration of Helsinki^15^.

**Figure 1:**
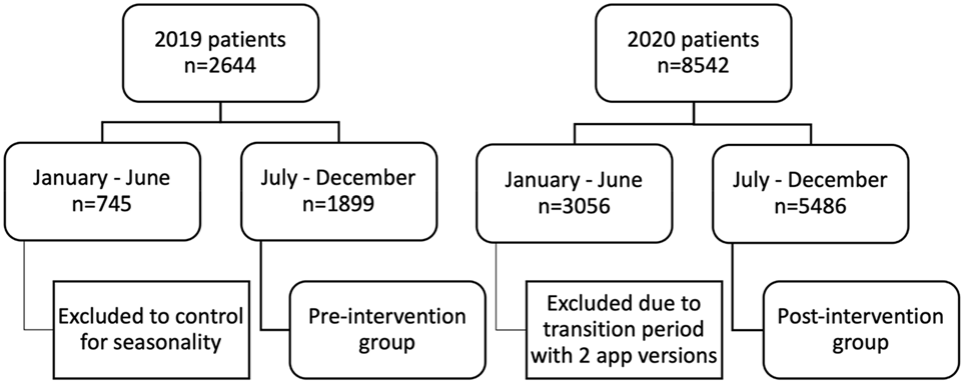
Flowchart of the included groups.

Data of all patients aged 18–64 years that (based on one intraoral scan) finished their treatment successfully with the so-called 1–1-2 system^14^ (see subsection: "Intervention and data collection") during the second half of 2019, i.e., never received e-reminders or e-feedback during their therapy (n = 1899 patients) and the second half of 2020, i.e., always received e-reminders and e-feedback (n = 5486 patients) were available. The introduction of e-reminders and e-feedback was the only difference between the two versions of the mobile application. A comprehensive sample was drawn (i.e., no random sampling, etc.). The patients showed the following characteristics prior to therapy: Malocclusion in the anterior and/or premolar region to be treated with CAT; adults (> 18 years) with a permanent dentition; absence of active periodontal disease or local and/or systemic conditions that can affect bone metabolisms; no extractions being required for the orthodontic treatment (Fig. 1).

### Intervention and data collection

A new e-reminder and e-feedback had been implemented and deployed to the PlusDental mobile application in the first quarter of 2020, reminding patients towards the 22 h aligner wearing time for ensuring tooth movement and instructing them to remove aligners only for eating, drinking, and oral hygiene. If patients recorded a wearing time of less than 22 h per day (they were actively asked to record this when changing aligners), they were sent an automatic e-feedback and given background information to increase the wearing time with the next aligner as “important information”.

All patients underwent standardized clinical and laboratory processes, as described in detail by Timm et al.^14^. At the first visit, a complete clinical examination, a full set of digital photographs and an intraoral scan as well as radiographs according to the recommendations of the British Orthodontic Society were performed^16^. A basic periodontal examination^17,18^ and a CMD screening^19^ were carried out in order to rule out contraindications to CAT. Eventually, patients received their set of aligners required for CAT. The aligners were trimmed 2 mm above the gingival margin and the 1–1–2 treatment protocol, consisting of 3 consequent aligners per step (0.5 mm for one week, 0.625 mm for one week, then 0.75 mm for 2 weeks), was followed. At this visit, patients were informed about the importance of wearing the aligners for 22 h per day and were instructed to check-in every aligner change using the mobile app (including, as described, recording of the wearing time).

A set of standardized intra- and extra-oral photographs were uploaded to the mobile application by patients at least every two months for a follow-up to ensure close monitoring of treatment and to encourage patient engagement.

### Outcome and outcome measures

The outcome in this study was self-reported patient compliance, recorded using the (application-based) questionnaire, measuring the daily aligner wearing time. Patients with consistent use of the mobile application for aligner check-in and an aligner wearing time of 22 h on 75% of their aligners were classified as fully compliant, as defined previously^14^. Patients with inconsistent application usage were classified as fairly or poorly compliant based on the aligner wearing time: Patients with the aligner wearing time of 22 h on 50–74.9% of their aligners were classified as fairly compliant and patients with an aligner wearing time of 22 h on only < 50% of their aligners as poorly compliant^14^.

### ITS Analysis

A semi-monthly cross-section of recorded patient poor compliance was employed for our ITS analysis. The data was partitioned by time, gender and age range (18 to 35, 36 to 55, and 56 to 64), resulting in 58 data points for the last six months of 2019 (pre-intervention cohort) and 64 data points for the last six months of 2012 (post-intervention cohort), as recommended for ITS^20^. Because it was found that the patient compliance was associated with a temporary slope change followed by a level change^21^ after the introduction of the intervention, the ITS was partitioned into three segments to account for this pattern: pre-intervention, post-intervention segment A (2020-07-01 to 2020-09-15) and post-intervention segment B (2020-10-01 to 2020-12-15). Since the outcome (poor compliance) is a count proportion, logistic regression was used for the analysis. The global model was as follows:

$$\begin{aligned} {\text{E}}\left[ {{\text{Y}}_{{\text{t}}} |{\text{ X}}_{{\text{t}}} } \right] \, & = {\text{ logit}} - {1}(\upbeta _{0} + \, \beta_{{1}} {\text{x time}}_{{\text{t}}} + \,\upbeta _{{2}} \times {\text{ segment}}\_{\text{A}}_{{\text{t}}} + \,\upbeta _{{3}} \times {\text{ segment}}\_{\text{A}}\_{\text{time}}_{{{\text{t }} + }}\upbeta _{{4}} \times {\text{ segment}}\_{\text{B}}_{{\text{t}}} \\ & + \,\upbeta _{{5}} \times {\text{ segment}}\_{\text{B}}\_{\text{time}}_{{\text{t}}} + \,\upbeta _{{6}} \times {\text{ gender}}\_{\text{m }} + \,\upbeta _{{7}} \times {\text{ age}}\_{36}\_{55 } + \,\upbeta _{{8}} \times {\text{ age}}\_{56}\_{64}) \\ \end{aligned}$$

where

- Y_t_ is the proportion of poor compliance among patients who were treated in each halfmonth t, X_t_ is the vector of the 8 covariates at time t, and E[Y_t_ | X_t_] is the expected value of Y_t_ given the covariates X_t_;
- time_t_ is a continuous variable that represents the time elapsed (in halfmonths) from the start of the observation period;
- segment_A_t_ is an indicator variable for whether halfmonth t was in post-intervention segment A (1 if in segment A, 0 otherwise);
- segment_A_time_t_ is a continuous variable giving the time elapsed (in halfmonths) within post-intervention segment A;
- segment_B_t_ is an indicator variable for whether halfmonth t was in post-intervention segment B (1 if in segment B, 0 otherwise);
- segment_B_time_t_ is a continuous variable giving the time elapsed (in halfmonths) within post-intervention segment B;
- gender_m is an indicator for gender (0 if female, 1 if male);
- age_36_55 is an indicator for age (1 if the age range is 36 to 55, 0 otherwise);
- age_56_64 is an indicator for age (1 if the age range is 56 to 64, 0 otherwise);
- β_0_ is an estimate of the log of the baseline odds; and
- each β_i_ is an estimate of the log of the odds ratio of the respective covariate.

Some additional explanation of the last two points is given: The odds of an event with probability p is the ratio p / (1 – p). In this case p is the probability of a patient being poorly compliant. If all the covariates are 0, then the estimated odds of poor compliance are exp(β_0_). The other coefficients relate to odds ratios. For instance, exp(β_6_) is an estimate of the ratio of the odds given gender_m = 1 to the odds given gender_m = 0 (with all other covariates equal), i.e. exp(β_6_) is a measure of how more/less likely male patients are to be poorly compliant than female patients.

The methodology proposed by Burnham & Anderson^22^ was used, in particular multimodel inference (MMI). The essential idea is to average over different models, rather than attempting to select a single best model. The key ingredient is the Akaike information criterion (AIC), a measure of relative model quality founded on information theory. To this end, all submodels of the global model were fitted to the data; that is, for each of the 256 subsets of {β_i_ : 1 ≤ i ≤ n}, a model using only the selected covariates was fitted to the data. (For brevity we conflate covariates with their coefficients in the model.) The AIC of each model was calculated. Note that the small-sample version of the AIC (AICc) was not used: Although the data seemingly consists of only 122 (= 58 + 64) points, each of these points represents a group of patients. Indeed, the number of patients was used to weight the points in the model fitting, which is in fact equivalent to fitting a logistic regression model on the level of individual patients, with a response of either 1 (poorly compliant) or 0 (not poorly compliant). For each model, the difference ∆ in its AIC from the smallest AIC was calculated (so the top model has ∆ = 0). These differences were exponentiated and then normalised to give the Akaike weight of each model. A subset of models was chosen on the basis of the Akaike weights: The models {β0, β2, β3, β4, β6} and {β0, β2, β3, β4, β6, β8} had Akaike weights of 0.196 and 0.168 respectively, while the next highest weight was 0.099. These two models were thus selected and their Akaike weights renormalized. (Other selection criteria were explored, such as ∆ < 2 or the smallest subset such that the sum of the Akaike weights was above 0.95, but each resulting averaged model suffered from multicollinearity amongst the covariates and in particular a non-positive-definite estimated covariance matrix.) The resulting model, based on weighted averages over the selected models, is henceforth referred to as the MMI model.

The descriptive data analysis and pairwise testing were performed using JASP 0.14.1 (University of Amsterdam, Amsterdam, The Netherlands). The ITS analysis was performed using version 0.13.1 of the Python library statsmodels^24^. The pre-and post-intervention groups were pairwise compared with a two-sided Chi-square test or a t-test for independent samples. *p* values less than 0.05 were considered statistically significant.

## Results

### Cohort characteristics

Data of 5486 patients were available for analysis; of these, 1615 (29.4%) were male and 3871 (70.6%) females. The median age at treatment initiation was 28 (range 18–64) years, the mean age was 29.1 (range 18–64) years. The largest age group were young adults (aged 18–35 years, n = 4456), followed by adults (aged 36–55 years, n = 984) and older adults (aged > 55 years, n = 46). While the mean age was not significantly different between pre-and post-intervention periods, there was a significant gender and age group distribution difference (*p* < 0.05) between the cohorts (Table 1).

**Table 1:** Cohort characteristics, stratified into pre-and post-intervention periods.

Covariates	Pre-intervention	Post-intervention	*p* value
No. of subjects (n)	1899	5486	
Age (mean ± SD)	28.8 ± 7.4	29.1 ± 8.1	0.13
Gender, female / male (%)	75.6% / 24.4%	70.6% / 29.4%	< 0.001
Age 18- to 35- years old (%) Age 36- to 55- years old (%) Age 56- to 64- years old (%)	1601 (84.3%) 286 (15.1%) 12 (0.6%)	4456 (81.2%) 984 (17.9%) 46 (0.8%)	< 0.05

### Compliance

Patients were classified according to the compliance criteria into full, fair, and poor compliance. Pre-intervention, 703/1899 (37.0%) patients showed full compliance, 729/1899 (38.4%) fair compliance, and 467/1899 (24.6%) poor compliance, whereas post-intervention 2382/5486 (43.4%) of patients showed full compliance, 2262/5486 (41.2%) fair compliance, and 842/5486 (15.3%) poor compliance. Compliance was higher in males than females (p < 0.001). No significant difference in compliance was found between age groups (Table 2).

**Table 2:** Compliance in the pre-and post-intervention cohorts, stratified by age group and by gender. A Chi-square test was used to test for differences in the strata in each cohort.

	Pre-Intervention					Post-Intervention				
	Overall sample	Full compliance	Fair compliance	Poor compliance	Chi-square	Overall sample	Full compliance	Fair compliance	Poor compliance	Chi-square
Male	463 (24.4%)	185 (9.7%)	197 (10.4%)	81 (4.3%)	X^2^ (2, n = 1899) = 16.72 *p* = 0.000233 (*p* = < 0.001)	1615 (29.4%)	763 (13.9%)	648 (11.8%)	204 (3.7%)	X^2^ (2, n = 5486) = 19.39 *p* = 0.000061 (*p* = < 0.001)
Female	1436 (75.6%)	518 (27.3%)	532 (28.0%)	386 (20.3%)		3871 (70.6%)	1619 (29.8%)	1614 (29.7%)	638 (11.7%)	
18- to 35- years old	1601 (84.3%)	588 (31.0%)	616 (32.4%)	397 (20.9%)	X^2^ (4, n = 1899) = 2.80 *p* = 0.591	4456 (81.2%)	1925 (35.1%)	1843 (33.6%)	688 (12.5%)	X^2^ (4, n = 5486) = 1.28 *p* = 0.864
36- to 55- years old	286 (15.1%)	112 (5.9%)	109 (5.7%)	65 (3.4%)		984 (17.9%)	438 (8.0%)	401 (7.3%)	145 (2.6%)	
56- to 64- years old	12 (0.6%)	3 (0.2%)	4 (0.1%)	5 (0.3%)		46 (0.8%)	19 (0.3%)	18 (0.3%)	9 (0.2%)	
Total	1899 (100%)	703 (37.0%)	729 (38.4%)	467 (24.6%)		5486 (100%)	2382 (43.4%)	2262 (41.2%)	842 (15.3%)	

Given that gender, but not age group distribution was significantly associated with compliance, we further compared compliance in both cohorts stratified by gender (Table 3). We confirmed that compliance was significantly higher in the post-intervention than the pre-intervention group in both genders.

**Table 3:** Compliance within gender groups pre-and post-intervention. A Chi-square test was used to test for differences between intervention groups.

	Full Compliance	Fair Compliance	Poor Compliance	Chi-Square
**Female**				
Pre-intervention	518 (36.1%)	532 (37.0%)	386 (26.9%)	X^2^ (2, n = 5307) = 72.89 *p* = < 0.0001
Post-intervention	1619 (41.8%)	1614 (41.7%)	638 (16.5%)	
**Male**				
Pre-intervention	185 (40.0%)	197 (42.5%)	81 (17.5%)	X^2^ (2, n = 2078) = 10.91 *p* = 0.0042 (< 0.05)
Post-intervention	763 (47.2%)	648 (40.1%)	204 (12.6%)	

**Figure 2:**
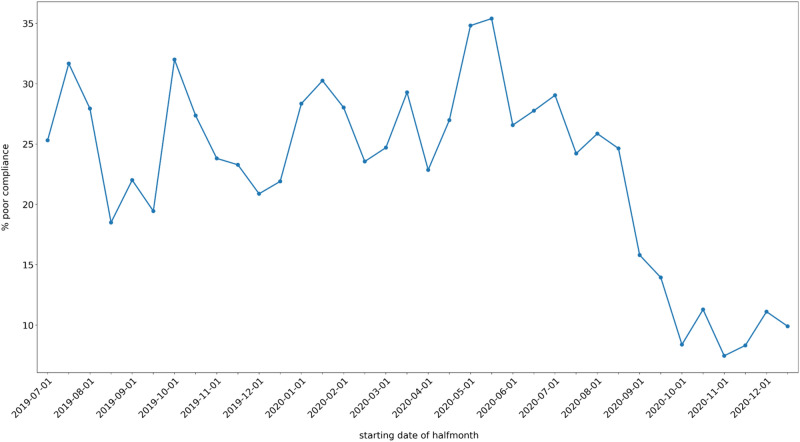
Mean poor compliance by starting date of treatment. Note that this figure contains data from the mid-period (January to June 2020), which was excluded from the statistical analysis.

**Figure 3:**
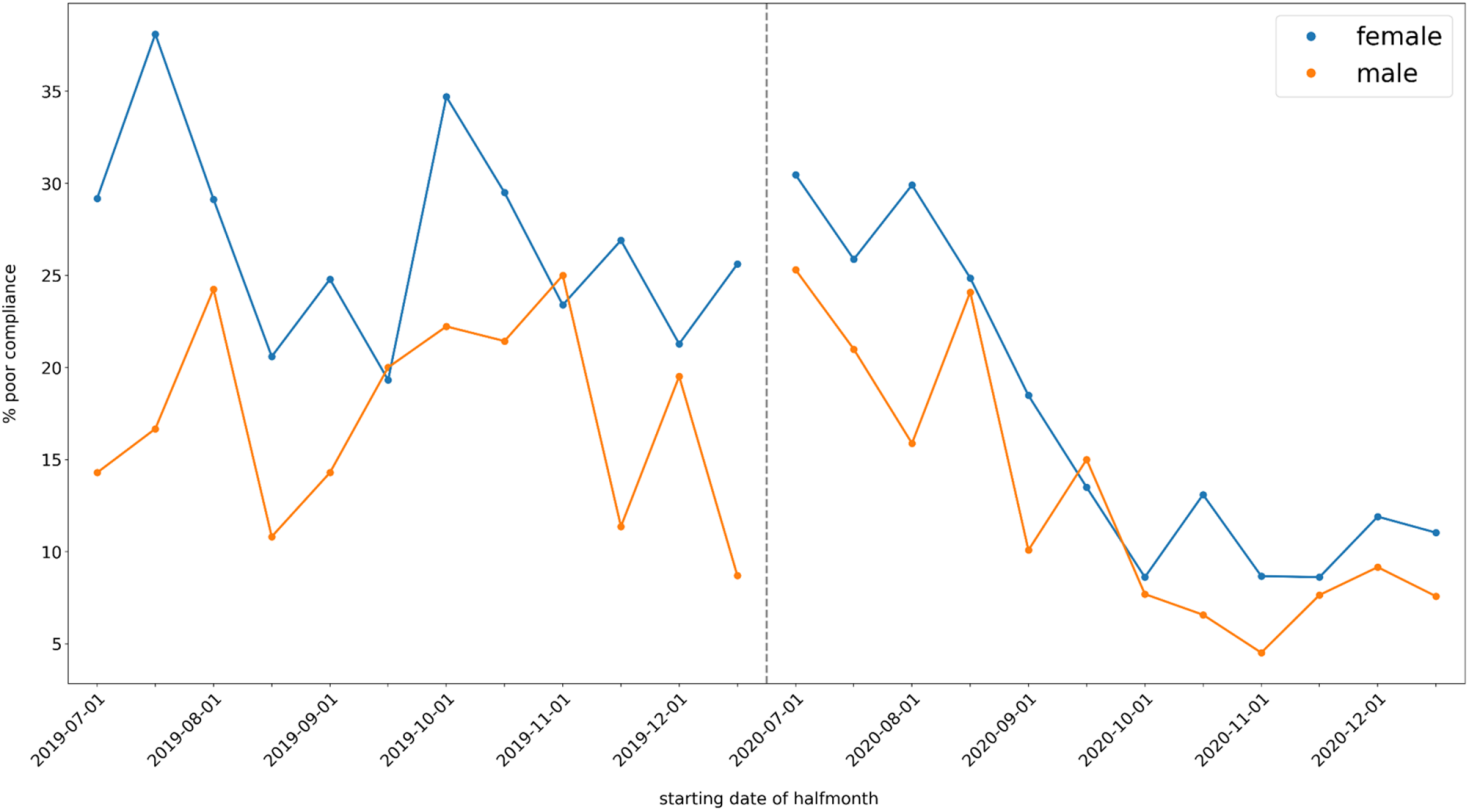
Mean poor compliance by starting date of treatment, split by gender. The dashed vertical line indicates the gap between the two segments.

**Figure 4:**
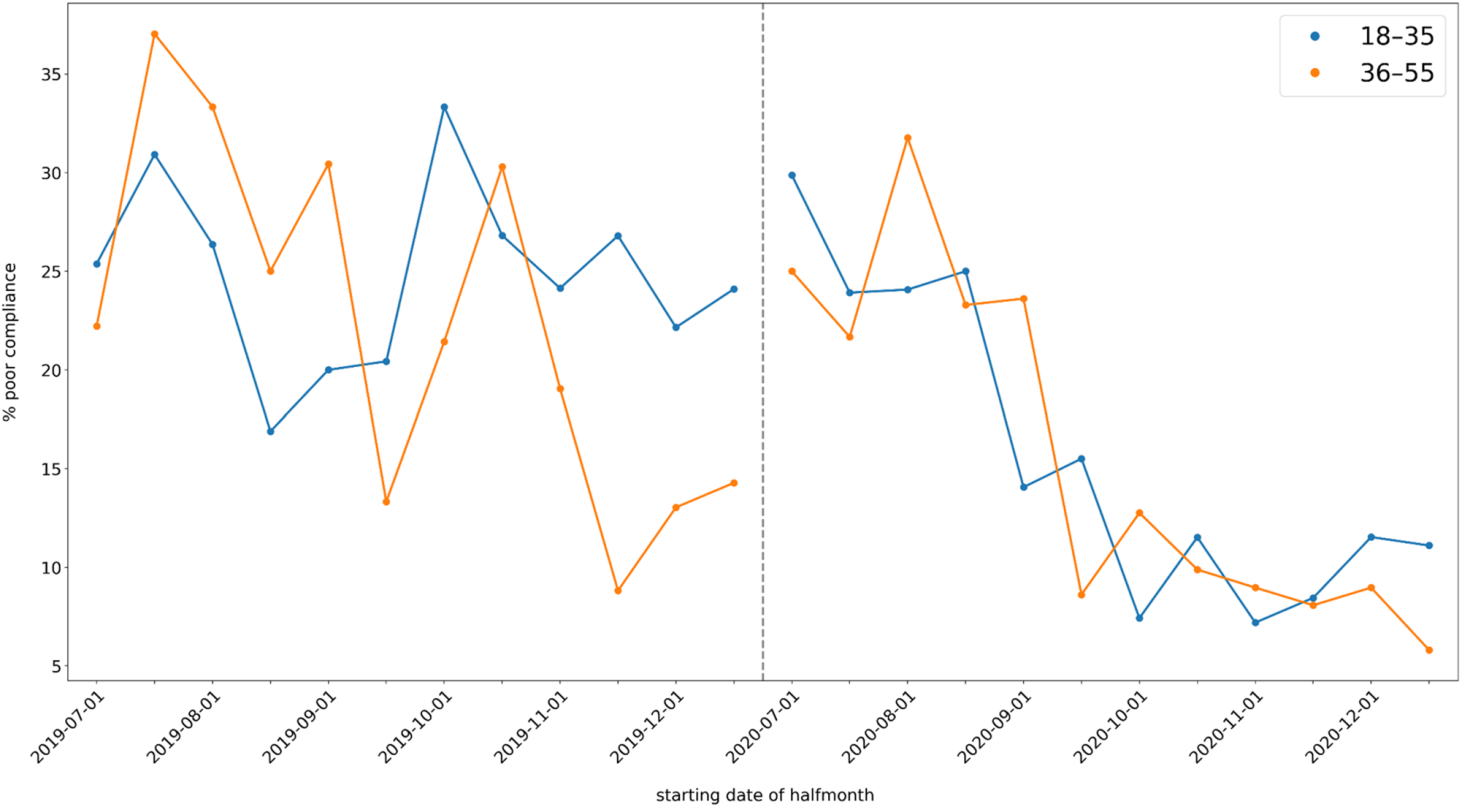
Mean poor compliance by starting date of treatment, split by age range. The dashed vertical line indicates the gap between the two segments. The age range 56–64 was excluded from the figure due to the low number of patients in this age group.

**Figure 5:**
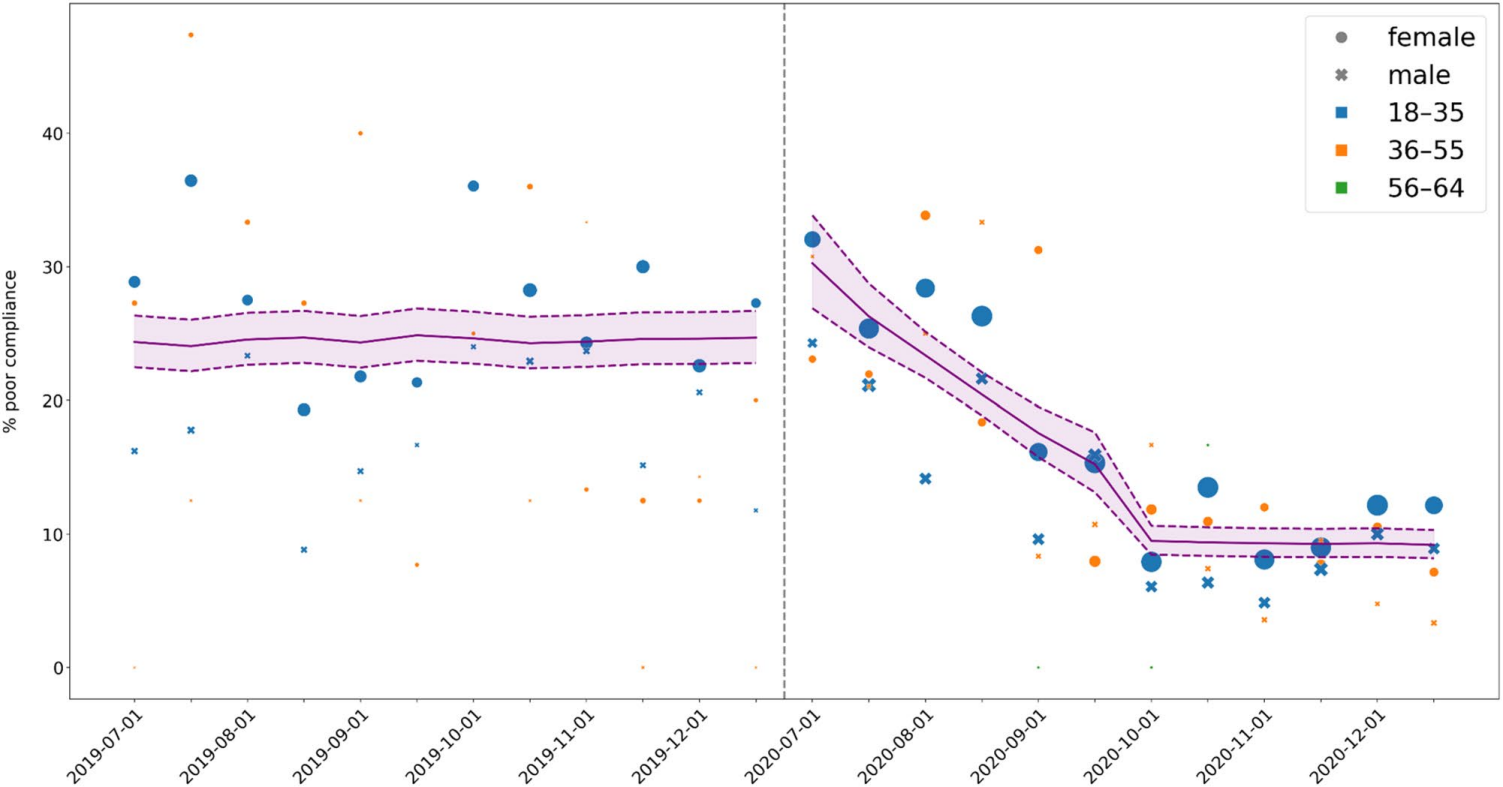
The MMI model by starting date of treatment. The purple line gives the point estimates of expected poor compliance while the purple shaded area is the 95% confidence band. Each point indicates the mean poor compliance of the given group, whereby the size of the point is proportional to the number of patients contained within that group. Points with fewer than 5 patients were excluded from the figure. The dashed vertical line indicates the gap between the two segments.

Descriptive plots of poor compliance over time are given in Figs. 2, 3 and 4. The coefficients of the MMI model are shown in Table 4. Note that p-values are not given, since doing so would conflate different analysis paradigms; instead, the Akaike weights of the coefficients can be used as a measure of the relative strength of evidence. Figure 5 shows the MMI model by starting date of treatment. The values were calculated by taking the mean of each covariate at the given starting date and then using the coefficients in Table 4 to make a point estimate. The 95% confidence band was calculated using the estimated covariance matrix of the MMI model (not shown). Similarly, the figures given in the Abstract—24.47% (95% CI: 22.59% to 26.46%) poor compliance in the pre-intervention period and 9.32% (95% CI: 8.31% to 10.45%) in post-intervention period segment B—were calculated by taking the mean of each covariate within each segment and using these as inputs for the MMI model. The results of the MMI model suggest that the introduction of e-reminders had the desired effect, namely reducing poor compliance. Note in Table 4 that the Akaike weight for age_55_64 is lower than the weights of the other coefficients. Moreover, the sign of coefficient is not stable over the 95% confidence interval. Both of these points are consistent with the findings of the chi-squared test that age was not significantly associated with compliance.

**Table 4:** The coefficients of the MMI model.

	Coefficient	95% confidence interval	Akaike weight
Constant term (β_0_)	− 1.03	(− 1.14, − 0.92)	1.00
Segment A (β_2_)	0.31	(0.11, 0.50)	1.00
Segment A time (β_3_)	− 0.35	(− 0.46, − 0.24)	1.00
Segment B (β_4_)	− 1.13	(− 1.29, − 0.97)	1.00
Gender_m (β_6_)	− 0.40	(− 0.55, − 0.26)	1.00
Age_56_64 (β_8_)	0.42	(− 0.19, 1.04)	0.46

Over the pre-intervention period, no significant difference or trend in compliance was identified (Fig. 5). In comparison, after the introduction of the e-reminders and e-feedback (i.e., post-intervention), the percentage of poorly compliant patients decreased substantially and remained consistently and significantly lower than pre-intervention. The downward trend did not continue indefinitely; Fig. 5 shows that the trend levelled off in October 2020, i.e., a temporary slope was followed by a level change as described. Note that the initial increase in post-intervention segment A is consistent with a seasonal effect since both July 2019 and July 2020 saw peaks in poor compliance.

## Discussion

This is the first ITS study using real-world clinical data to investigate the impact of using e-reminders and e-feedback via a mobile application during CAT. Our results showed that the introduction of e-reminders and e-feedback significantly and sustainably improved compliance. While compliance was generally higher in males than females, the positive effect of the intervention was found in both males and females alike. Orthodontists and dentists may want to employ mobile reporting options (like apps) to identify low compliance in CAT patients and to address this low compliance using e-reminders.

Although a randomized controlled trial is considered the gold standard in attempting to prove causality and to demonstrate the effectiveness of a new intervention, such trials are not always feasible nor needed^25,26^. For instance, the running costs and time required for conducting trials, the associated bias by selection, and attrition are among the limitations of randomized controlled studies^26–28^. In our study, we instead opted for an ITS design, which is one of the strongest quasi-experimental designs^26^ not requiring randomization and having been found to have high internal validity^20,21,29,30^. Using the ITS approach on routine data allowed to demonstrate the effects of the e-intervention on compliance in a large real-life population, i.e., with high generalizability and applicability.

In this study among CAT patients, we analysed factors influencing patient compliance and trends before and after an intervention with e-reminders that emphasized the importance of compliance in wearing time. Since the intervention (a new version of the mobile application, now including e-reminders, etc.) was deployed during the first quarter of 2020, the period between January 2020 and June 2020 included both patients who had started their treatment with the old app version and those starting with the new one; this period was therefore excluded to ensure consistency. Based on that and to control for seasonality, the period from January 2019 to June 2020 was excluded as well.

Although the gender and age group distribution differed significantly between the pre-intervention and post-intervention groups, we did not find this to impact on compliance: Age was not significantly associated with compliance, and the combined effect of gender and intervention on compliance was analysed accordingly using stratification analysis. We found the proportion of individuals with poor compliance to decrease significantly in both males and females after the introduction of the e-reminders and e-feedback.

To our knowledge, there is no published ITS study investigating the effect of an intervention on patient compliance during CAT, nonetheless, the findings can be compared to a range of other reports. Reminders have been found to have a positive effect on compliance during orthodontic treatment based on the results of two systematic reviews, which is in line with our study^8,31^. Our findings are also in agreement with a randomized trial showing significantly increased levels of oral hygiene compliance in orthodontic patients that received verbal education on their treatment during their first visits^7^. Added to that, the positive effect of active reminders established in our study is also similar to the finding by Eppright et al., where weekly text message reminders highlighting the importance of oral hygiene were shown to improve oral hygiene compliance in orthodontic patients^6^. Moreover, the effect of regular positive reinforcement on general oral health through text messaging established by Jadhav et al. is also in agreement with our findings^13^.

Among many methods, reminding the patient on a regular basis to adhere to the treatment protocol has been demonstrated to increase compliance before^32,33^. Patients found the mobile applications easy to use and demonstrated the ability to use the app^34,35^, preferred the smartphone app intervention to other devices^35^, and were satisfied with in-app functions that promote patient compliance with e-reminders^36^. The tendency to overestimate one’s own wearing times is reduced once patients know that the wearing time is monitored^37^. In addition to sending e-feedback and e-reminders to patients on regular basis, financial incentives were reported to enhance patient compliance^38^. In the context of the mobile application, a gaming component could be considered to further optimize compliance^39,40^. The efficacy of these methods in CAT patients needs to be further studied.

This study has a number of strengths and limitations. First, it is one of the first reports to demonstrate the effect of e-reminders on improving patient compliance during CAT treatment. This finding is particularly significant as the literature indicates that orthodontic patients seeking treatment with removable appliances often fail to adhere to wearing times^41–43^ or sometimes do not complete the prescribed therapy at all^44^. Another strength is that it used real-world data and employed the ITS approach. By doing so, high generalizability and applicability, as well as a relatively large sample size, were combined with high internal validity. Among the limitations of this study is the exclusion of the January 2020 to June 2020 group due to the impracticability of deploying the intervention for all the patients at the same time. However, as with many interventions, a transition period is needed for the intervention to produce the anticipated effect. This lag period could be excluded from the analysis to avoid faulty interpretation of the analysis^20^. The ITS analysis also had limitations: The decision to partition the data into three segments was post-hoc, although it was necessary to correctly model the data since a two-segment approach cannot reflect a temporary slope followed by a level change. Moreover, it would have been useful to have a longer pre-intervention time period to build on more time points and capture possible patterns of seasonality and differentiate them from outliers. A longer pre-intervention period may also help to alleviate issues with multicollinearity (see Sect. ITS Analysis). As a result, and to tackle the aspect of seasonality, we only included individuals over similar time periods in 2019 (pre-intervention) and 2020 (post-intervention). We showed that during summer, compliance decreased, possibly due to different cognitive distractions during periods of good weather^45^. Another limitation is that the aligner wearing hours were reported by the patients using self-reports, which are susceptible to biases. More in-depth data analyses and, possibly, qualitative investigations are needed to address these limitations in detail.

## Conclusion

Within the limitations of this study, e-reminder and e-feedback were effective measures for increasing compliance in CAT patients. Orthodontists and dentists may consider digital monitoring and e-reminders to improve compliance and thereby increase treatment success.

## Data Availability

The data is available upon request to the corresponding author.
